# Treating gaps and biases in biodiversity data as a missing data problem

**DOI:** 10.1111/brv.13127

**Published:** 2024-08-08

**Authors:** Diana E. Bowler, Robin J. Boyd, Corey T. Callaghan, Robert A. Robinson, Nick J. B. Isaac, Michael J. O. Pocock

**Affiliations:** ^1^ UK Centre for Ecology & Hydrology Maclean Building Benson Lane Wallingford OX10 8BB UK; ^2^ Department of Wildlife Ecology and Conservation, Fort Lauderdale Research and Education Center University of Florida 3205 College Avenue Davie Florida 33314‐7719 USA; ^3^ British Trust for Ornithology The Nunnery Thetford Norfolk IP24 2PU UK

**Keywords:** biodiversity change, citizen science, ecological modelling, macroecology, spatial bias

## Abstract

Big biodiversity data sets have great potential for monitoring and research because of their large taxonomic, geographic and temporal scope. Such data sets have become especially important for assessing temporal changes in species' populations and distributions. Gaps in the available data, especially spatial and temporal gaps, often mean that the data are not representative of the target population. This hinders drawing large‐scale inferences, such as about species' trends, and may lead to misplaced conservation action. Here, we conceptualise gaps in biodiversity monitoring data as a missing data problem, which provides a unifying framework for the challenges and potential solutions across different types of biodiversity data sets. We characterise the typical types of data gaps as different classes of missing data and then use missing data theory to explore the implications for questions about species' trends and factors affecting occurrences/abundances. By using this framework, we show that bias due to data gaps can arise when the factors affecting sampling and/or data availability overlap with those affecting species. But a data set *per se* is not biased. The outcome depends on the ecological question and statistical approach, which determine choices around which sources of variation are taken into account. We argue that typical approaches to long‐term species trend modelling using monitoring data are especially susceptible to data gaps since such models do not tend to account for the factors driving missingness. To identify general solutions to this problem, we review empirical studies and use simulation studies to compare some of the most frequently employed approaches to deal with data gaps, including subsampling, weighting and imputation. All these methods have the potential to reduce bias but may come at the cost of increased uncertainty of parameter estimates. Weighting techniques are arguably the least used so far in ecology and have the potential to reduce both the bias and variance of parameter estimates. Regardless of the method, the ability to reduce bias critically depends on knowledge of, and the availability of data on, the factors creating data gaps. We use this review to outline the necessary considerations when dealing with data gaps at different stages of the data collection and analysis workflow.

## I. INTRODUCTION: UNEVEN SAMPLING OF BIODIVERSITY

Ecologists have ever‐growing access to data on species' occurrence and abundances. Potential sources of data include long‐term citizen‐science monitoring schemes (such as the North American Breeding Bird Survey) (Bled *et al*., 2013), data aggregators [such as the Global Biodiversity Information Facility (GBIF)] (Garcia‐Rosello *et al*., 2015), remote‐sensing platforms (Fretwell, Scofield & Phillips, 2017) and synthesis databases (such as BioTIME or the Living Planet Database) (Dornelas *et al*., 2014). Since these data cover broad spatial and temporal scales, they are especially useful for large‐scale questions, for instance, about species' distributions, population and community‐level trends, and ecological niches (Chandler *et al*., 2017; Sullivan *et al*., 2017; Fink *et al*., 2020). These data also underpin many biodiversity trend indicators that are central for national and international conservation policy (Gregory *et al*., 2005; van Swaay *et al*., 2008; Fraisl *et al*., 2020).

Despite the impressive volume of data, biodiversity data, regardless of the source, tend to contain gaps (Boakes *et al*., 2010). Data gaps are not necessarily a problem; indeed, most ecological studies rely on statistical inference to make inferences about a broader region of interest from a sample. Data gaps, however, can be problematic when they lead to biases (Boakes *et al*., 2010; Bled *et al*., 2013; Amano, Lamming & Sutherland, 2016). Many ecologists have raised concerns about the impacts of bias due to data gaps on estimated spatial or temporal biodiversity patterns (Bayraktarov *et al*., 2019; Valdez *et al*., 2023). For instance, biases could mean that species' trends are over‐ or under‐estimated, leading to ill‐informed decisions about which species should be conservation priorities and misplaced direction of conservation action. Developing methods to deal with data gaps and associated biases within large‐scale biodiversity data is an increasingly important task to make full use of the growing big data sources.

Patterns in the availability of biodiversity data are affected by the original motivations for, and constraints on, data‐collection, reporting and mobilisation activities. There are, however, typical patterns in data availability that indicate common causes of data gaps. Spatial patterns in the data available from citizen science, which form the majority of monitoring data (Chandler *et al*., 2017), have been especially well studied. Citizen‐science programs have varying degrees of standardisation in protocol and sampling designs (Isaac & Pocock, 2015; Pocock *et al*., 2017) but more data are typically collected in accessible areas such as near roads and urban areas, leading to data gaps in remote areas (Geldmann *et al*., 2016). Such biases are not unique to citizen‐science data, as even data collected during formal scientific studies have potential sampling biases; for instance, towards regions undergoing less habitat change (Gonzalez *et al*., 2016; Forister *et al*., 2023; Cardinale *et al*., 2018). Various solutions have been proposed to deal with these biases (Hefley *et al*., 2013; Cretois *et al*., 2021; Johnston *et al*., 2020; Ver Hoef *et al*., 2021), but there is still a lack of a general framework for ecologists to guide decisions on when and how to deal with data gaps.

Here, we show how using missing data theory (Rubin, 1976) can unify problems associated with data gaps across different types of biodiversity data sets. Missing data are a widespread problem crossing disciplines, with a large body of literature on their implications and possible solutions (Little & Rubin, 2019; Carpenter & Kenward, 2012; Carpenter & Smuk, 2021). We expect that aligning the generalised problem of missing data, conceptualised within missing data theory, to the problem of biodiversity data gaps discussed above will yield opportunities so far overlooked. We mostly focus our review on modelling trends in species occupancy or abundance using monitoring data collected by volunteer citizen scientists, but the ideas transfer to other types of biodiversity data or questions. The general problems and potential solutions could be applied to animal or plant data, or terrestrial or marine systems; however, the implications of ignoring data gaps, and the ability to account for problematic data gaps, will vary according to the causal factors at play in both sampling probability and biodiversity patterns and the availability of data to model them. We show that bias is not a property of a data set; rather, bias is a property of the use of a data set for a specific question and target population that are imposed by the data analyst. We review some commonly used solutions for missing data to highlight potential approaches that could be considered in biodiversity analyses.

## II. CLASSIFYING DATA GAPS USING MISSING DATA THEORY

### (1) Biodiversity data gaps

**Fig. 1:**
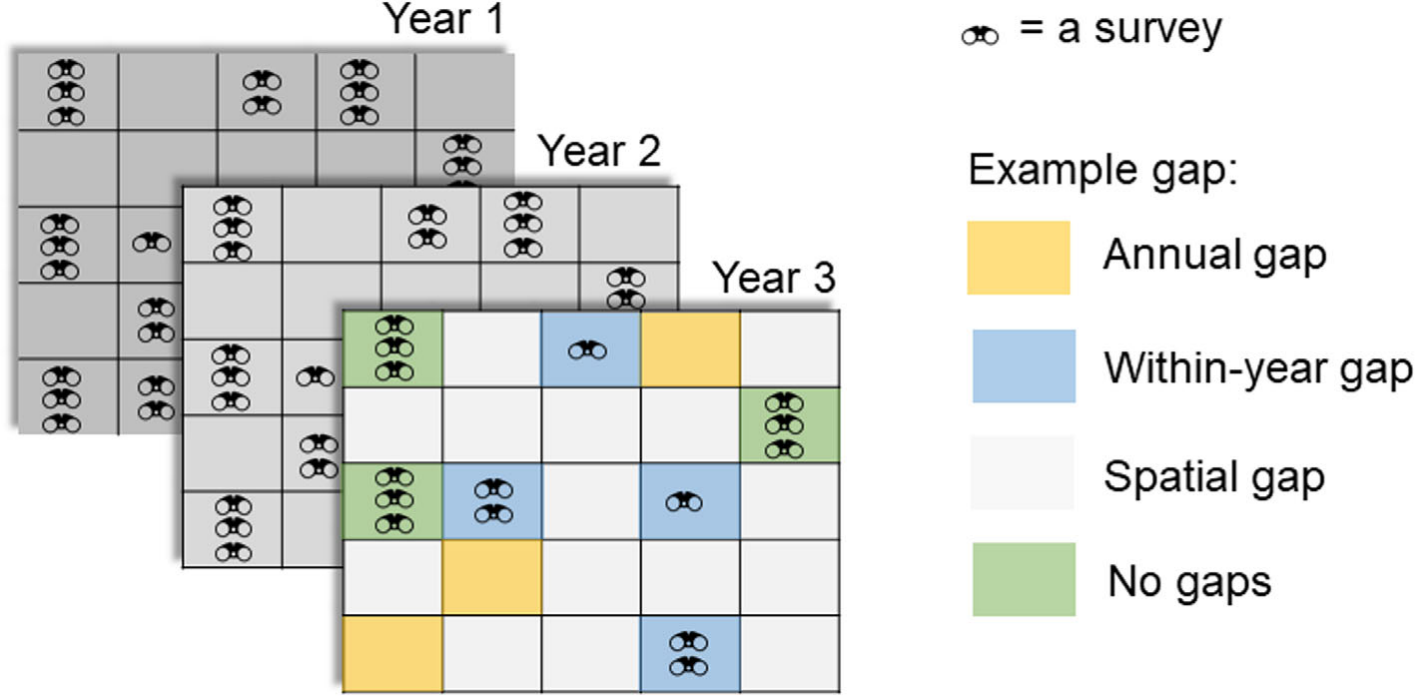
Different types of data gaps within biodiversity data. We imagine a scenario where there are multiple survey visits across sites and years. Visits can be in response to a protocol (“structured” data) or opportunistic (“unstructured”), and repeat visits can be made by the same or different recorders. Data gaps, or more generally uneven data availability, can arise due to (*a*) within‐year gaps (e.g. blue squares, i.e. ordinarily there are three visits, but some sites are only visited once or twice in a year), (*b*) annual gaps (e.g. yellow squares, i.e. some sites that are usually sampled are entirely unvisited in some years) or (*c*) spatial gaps (e.g. light grey squares, i.e. some sites within the region of interest are never visited across all years). Some sites are well‐sampled within and across years and hence have no missing data (e.g. green squares).

Species occurrence or abundance data can have gaps in different dimensions. We distinguish between spatial, annual and within‐year gaps (Fig. 1). We define spatial gaps as those formed by sites with no data, and annual gaps as those formed by a lack of data in some years at sites that otherwise have been sampled. Together, spatial and annual gaps determine the spatial and temporal coverage of a data set. Within‐year gaps arise when data are lacking in specific seasons or months, which can be important because most organisms are seasonal and multiple visits can be used to estimate detection probabilities. Biodiversity data sets can also have taxonomic gaps (Troudet *et al*., 2017) – this is outside the scope of this review since we are primarily interested in the implications of data gaps for species‐level questions asked by monitoring schemes. However, some of the approaches we discuss later for dealing with spatio‐temporal gaps have been applied to account for taxonomic gaps (e.g. weighting in the Living Planet Index, McRae, Deinet & Freeman, 2017) and missing data thinking could be extended to these types of gaps.

Considering why these gaps arise can help understand their likely impact, for instance, on species long‐term trend estimation. Data gaps can be found in all types of monitoring data including highly structured monitoring schemes with a standardised protocol, such as many national bird survey schemes, as well as unstructured/opportunistic monitoring data that are typically an aggregation of heterogeneous observations. While both structured and opportunistic monitoring data can be affected by similar data gaps (Binley & Bennett, 2023), there are some key differences between these types of monitoring data. In structured schemes with a formal spatial sampling design, data gaps include both planned and unplanned gaps. Planned gaps arise because only a sample of sites was ever intended to be sampled. Unplanned gaps can still occur in these schemes, for instance due to a failure to recruit and retain surveyors at sites that were intended to be sampled (Zhang *et al*., 2021; Marsh & Cosentino, 2019). In most other types of data and monitoring schemes, there is no large‐scale planned spatial sampling design. Some monitoring schemes have sampling protocols but participants are free to choose their own sampling sites. In fully opportunistic monitoring schemes, participants make individual decisions about where to sample and gaps emerge from unevenness in the cumulative sampling effort of all participants. Due to the high number of participants, and lack of coordination of their effects, sampling effort is generally more strongly skewed across space and time in opportunistic schemes than in structured schemes, leading to more pervasive data gaps (Geldmann *et al*., 2016). Synthesis databases such as BioTIME and the Living Planet Database, and data aggregators such as GBIF, are similar in these respects to schemes without a spatial sampling design since they contain data that were independently collected as part of separate studies, without any coordination in their efforts.

Drivers of data gaps may differ across data sets because of differences in sampling objectives and constraints, but similar gaps are often found within monitoring schemes involving citizen scientists. Spatial gaps often occur in remote areas because there is a smaller pool of potential participants nearby (Geldmann *et al*., 2016; Mandeville, Nilsen & Finstad, 2022). Spatial gaps can also be more common where species have lower abundance or land cover is perceived to be less attractive for species and for surveying, e.g. agricultural land (Tulloch *et al*., 2013; Dambly *et al*., 2021; Marsh & Cosentino, 2019). At large scales, gaps can also be associated with socio‐economic variables such as metrics of economic activity that might be associated with lower sampling and data‐compilation effort (Meyer *et al*., 2015). Annual gaps can arise due to project turnover or because of external factors (e.g. the 2020 season for most countries was highly compromised by the Covid‐19 pandemic). Annual gaps have also been linked with local land use changes that negatively affected species abundance (Zhang *et al*., 2021; Marsh & Cosentino, 2019). Within‐year data gaps can be caused by periods of inclement weather (Zimney & Smart, 2022; Diekert *et al*., 2023) or vary seasonally, for example missing surveys for butterflies are more common at the start and end of the main flight period (Dennis *et al*., 2016), while bird sampling can be higher during their migration periods (La Sorte & Somveille, 2020).

### (2) Classes of missing data

Within the classic missing data theory, there are three classes of missing data or missingness (Missing Completely at Random, Missing at Random, Missing Not at Random), defined below, each with different consequences for bias (Table 1) (Rubin, 1976; Nakagawa & Freckleton, 2008; Little & Rubin, 2019). These classes vary in their missing data mechanism, which describes the relationship between the probability of missing data (or sampling effort in the monitoring context) and the values of other variables. Hefley *et al*. (2013) proposed viewing spatial biases in presence‐only data as a form of missing data. Here, we extend it more broadly across different types of biodiversity data.

**Table 1:** Missing data classes in biodiversity data, including examples and implications.

Missing data class	Typical meaning	Meaning in the context of biodiversity data	Examples	Typical implications
Missing completely at random (MCAR)	Missingness is independent of observed and unobserved variables	Sampling is independent of any covariates, or covariates that affect sampling probability are independent of those affecting biodiversity	Within‐year: Weekdays Annual/spatial: Completion of a fixed‐term project or retirement of a participant	Ignorable
Missing at random (MAR)	Missingness is associated with observed data but not any unobserved variables	Covariates that affect sampling probability are shared with those affecting biodiversity, but data are available on all these covariates and included in the analysis	Within‐year: Season (day of year) Annual: Urban development Spatial: Accessibility	Ignorable if the model includes all relevant covariates
Missing not at random (MNAR)	Missingness depends on unobserved variables or the missing values themselves	Sampling varies with biodiversity values or an unknown or unavailable covariate affects sampling and biodiversity	Within‐year/annual/spatial: Unknown factors causing variation in species activity/abundance that correlate with sampling effort	Non‐ignorable – the missing data mechanism might need to be modelled

Within the context of biodiversity data, missingness can be regarded as Missing Completely at Random (MCAR) if the factors affecting sampling, and causing missingness, are independent of those affecting species (Table 1). Under MCAR missingness, the observed data are effectively a random sample of the whole population, and the distribution of values of the biodiversity variable of interest are similar in sampled and non‐sampled sites or times. For instance, if sampling site selection is driven by human accessibility, but species distribution is primarily driven by climate, and if accessibility and climate are not correlated, then spatial data gaps would be MCAR. Within‐year gaps associated with weekdays, because many volunteers only have the necessary spare time to sample at the weekends (Evans & Day, 2002; Courter *et al*., 2013), or annual gaps associated with project turnover, are also examples likely to cause MCAR data gaps since such gaps are probably not associated with species occurrence or abundance (Table 1). In this case, missing data could reduce the precision of parameter estimates through reduced sample size but would not increase the bias.

When the factors affecting sampling effort are the same as, or correlated with, those factors affecting species, the missing data are not MCAR and can either be Missing at Random (MAR) or Missing Not at Random (MNAR). In both cases, there are systematic differences in the distribution of values of the biodiversity variable of interest between sampled and non‐sampled sites or times (Table 1). For instance, if road density affects both sampling probability and species abundance, then any spatial gaps associated with roads are not MCAR. Similarly, habitat degradation at a site could reduce both species abundance and participant retention in a citizen science scheme, creating an annual data gap that is not MCAR (Table 1), because the missing values are lower than the sampled values.

We can separate MAR and MNAR by borrowing from the “Rumsfeld Matrix”: MAR are effectively “known unknowns” while MNAR are “unknown unknowns”. The “known” for MAR is knowledge and availability of data on the shared covariates affecting sampling probability and the biodiversity variable. If data for these covariates are available, and included in the analysis, then the missing data are MAR. Hence, despite its name, MAR in biodiversity monitoring does not mean that sampling effort is randomly distributed in the landscape. Rather, it means that the covariates affecting sampling are known and that there are available covariate data to explain fully the differences between sampled and non‐sampled sites/times. If any of the relevant factors affecting sampling and species are unknown, unavailable or not modelled, the missing data become MNAR (Table 1). Hence, decisions of the analyst, and whether to collect and model the effects of a specific variable, can determine whether a data gap is MNAR or MAR (discussed more fully in Section III). MNAR may also arise when missingness is dependent on the missing values of the biodiversity variable itself, that is, if sampling effort directly depends on species occurrence or abundance.

Statistical tests can only partly help assess which form of missingness is most likely (Little, 1988). Analysis of relationships between data availability and observed covariates can point towards MAR if some relationships are significant. But a lack of any association, or an incomplete explanation of data gaps, could reflect MCAR or MNAR. Because MNAR is associated with unavailable data, it cannot be tested directly. Concerns about whether missingness in the biodiversity data is directly associated with the data's values could be explored if there is a related variable with available data (Wu, 2022). We argue that MCAR is unlikely in most biodiversity data because many variables that affect sampling probability (such as road density or human population density) are also likely to affect species. Even in schemes with a planned spatial design, a similar set of variables are likely to be associated with unplanned data gaps that arise from variation in participant recruitment or drop‐out. However, MCAR is still a useful concept as a null hypothesis and because it is the assumption made when no consideration is given to adjust for data gaps when using monitoring data to estimate species trends. Since gaps in biodiversity data are caused by a range of different factors, some gaps may be understandable by knowledge of the data collection process and/or with available environmental covariates, while other gaps may be harder to explain. This means that data gaps are unlikely to be entirely MAR or MNAR, but typically a mixture.

## III. IMPLICATIONS OF MISSINGNESS FOR ECOLOGICAL QUESTIONS

**Fig. 2:**
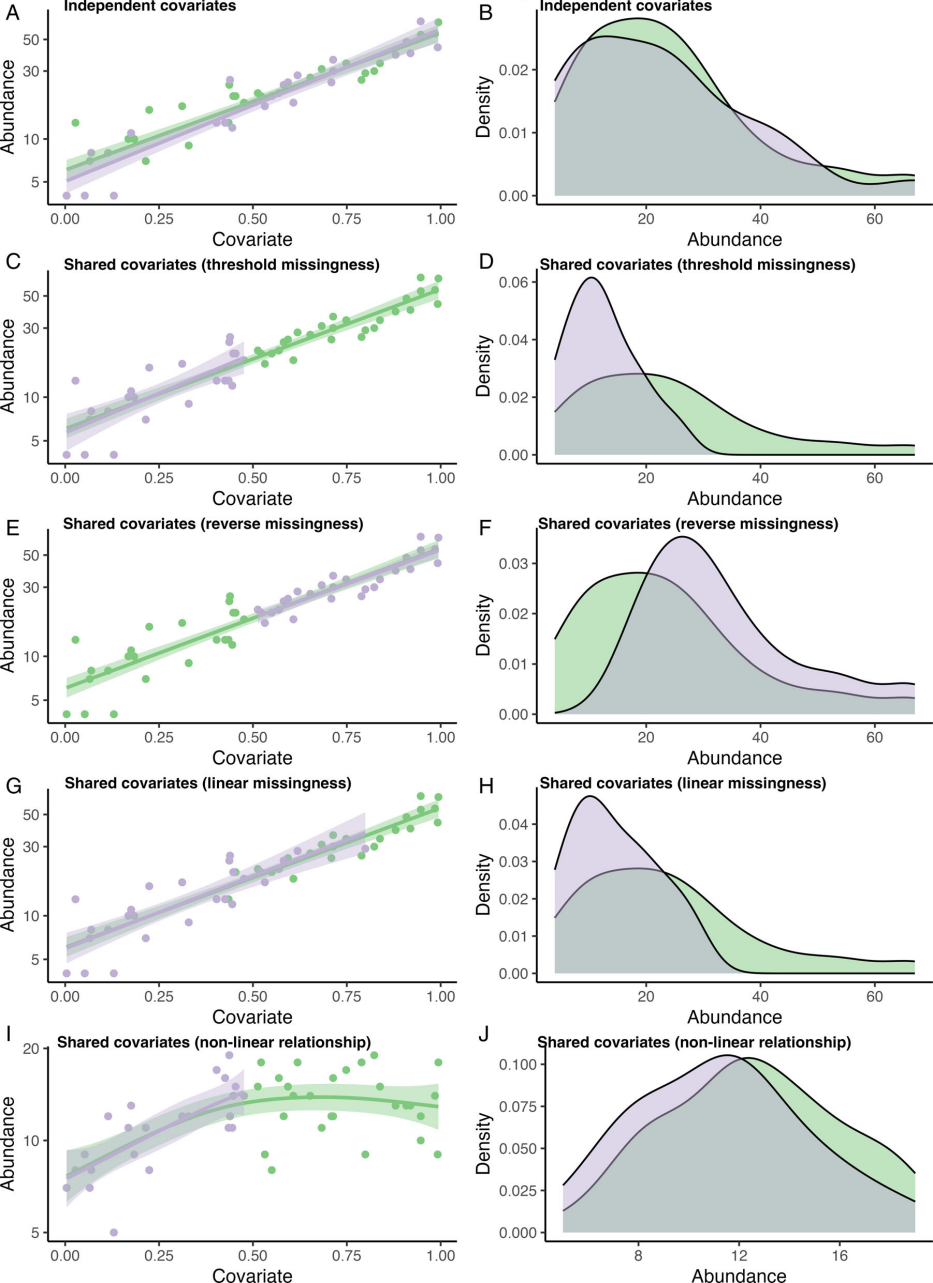
The impacts of different missing data patterns on regression (left) and sample distributions (right). We use a hypothetical data set to highlight different missing data mechanisms. In A and B, the covariate affecting sampling probability is *independent* from the covariate affecting species abundance. In this case, both the estimated effect of the covariate (e.g. in a linear regression, shown in A by the solid line) and the sample distribution (B) are similar in a data set with (purple) and without (green) missing data [i.e. missingness is “missing completely at random” (MCAR)]. In C and D, the covariate affecting sampling probability is the *same as or correlated with* the covariate affecting species abundance – in this case, data are missing when the covariate is above average (threshold missingness). The estimated effect of the covariate is the same in the data set with and without missing values (shown in C) but the sampling distribution is different (D). In E and F, the missingness pattern is reversed compared to C and D (i.e. data are missing when the covariate is below average), but we can similarly retrieve the same unbiased covariate effect (E) even though there is greater mean abundance in the data set with missing values (F). In G and H, the covariate affecting sampling probability is the *same as or correlated with* the covariate affecting species abundance – in this case, the probability of missing data increases with the value of the covariate (linear missingness rather than a theshold). Again, the estimated effect of the covariate is the same (shown in G) but the sampling distribution is different (H). In I and J, the covariate affecting sampling probability is the *same as or correlated with* the covariate affecting species abundance; additionally, the true relationship between the covariate and species abundance is non‐linear and data are missing when the covariate is above average.

Missing biodiversity data do not necessarily have strong impacts on the results of statistical modelling – the outcome often depends on the specific question and parameter of interest (Bartlett, Harel & Carpenter, 2015; Collins, Schafer & Kam, 2001; Little *et al*., 2022; Hughes *et al*., 2019). Viewing data gaps as a form of missing data can help decide whether a particular data gap matters. As we note above, data gaps that are MCAR do not cause bias, but data gaps in biodiversity data are unlikely to be MCAR. The “missing at random” assumption of MAR is conditional on accounting for variables affecting sampling probability within an analysis, require that these variables are known, reflected in available data and included in the analysis (Fig. 2) (Conn, Thorson & Johnson, 2017; Hefley *et al*., 2013). Because different ecological questions will lead to different decisions about which variables to collect and include in an analysis, a data gap might be MAR under some questions/analyses but MNAR under others. To illustrate these potential differences, we contrast two typical questions asked with biodiversity data.

### (1) Understanding the roles of environmental drivers on species' distributions

Monitoring data are often used to understand the environmental factors explaining species distribution patterns. The implications of missing data for species distribution models have been often considered in terms of niche truncation. Niche truncation happens when a data set only contains occurrence data from part of the geographic range of a species, which usually also means that the data set only covers part of the ecological/environmental space that is suitable for the species (Chevalier *et al*., 2022; Albert *et al*., 2010; Guo *et al*., 2023). These studies show that the implications of niche truncation depend on the functional form of the relationship between the associated covariate and the species response (Chevalier *et al*., 2022) and the type of monitoring data available (Baker *et al*., 2022).

We begin considering the scenario when abundance data are available. In this case, if there is a simple linear relationship between an environmental covariate and species abundance, missing data do not necessarily cause bias in the estimated effect of the covariate on abundance, even when missingness depends on the same covariate (Fig. 2A, C, E, G) (Collins *et al*., 2001). For instance, we could estimate the effect of elevation on species abundance without bias, even if elevation is associated with data gaps (e.g. if we are missing data from high‐elevation regions), provided elevation and abundance are linearly related. This is because the relationship between the covariate and species abundance can be estimated without bias using data over the sampled range of covariate values, as shown in Fig. 2C – the same relationship is found with a full data set (green in Fig. 2) or a restricted data set with data gaps (purple in Fig. 2). Missing data can, however, cause problems when the underlying relationship between the covariate and species abundance is non‐linear. In this case, data gaps hinder estimating the true form of the relationship (see Fig. 2I – the true curved relationship is fitted with the full data set but a simple positive linear relationship is fitted with the restricted data set). The fitted relationship using the restricted data set will critically depend on which portion of the covariate range is sampled. Since many ecological associations show some non‐linearity, or context dependencies such that relationships depend on the value of other variables (Spake *et al*., 2023), we expect this issue is likely to be widespread. We also note that we assumed a linear relationship on the log‐scale in our example (Fig. 2), which matched the log link function of the fitted regression model, but non‐linearity in other cases could also be affected by the specific link functions used in generalised linear models.

We now consider the alternative scenario of fitting a distribution model with presence‐only occurrence data, typical of opportunsitic citizen science. In this case, any data gaps within a geographic region could represent a lack of sampling or a lack of true species occurrence. This creates an inherent identifiability challenge for any model seeking to separate the sampling processes from the true ecological processes affecting species distributions (Hefley *et al*., 2013; Baker *et al*., 2022). Many methods have been developed to generate pseudo‐absences for analysis of presence‐only data (Barbet‐Massin *et al*., 2012; Hertzog, Besnard & Jay‐Robert, 2014), but they are still usually more prone to biases when there are shared covariates affecting sampling probability and true species occurrence (Baker *et al*., 2022). The target‐group background method is a popular approach to generate pseudo‐absences by integrating data from multiple species assumed to be surveyed by similar methods/people. With this method, the aim is to produce absence data with a similar pattern of spatial sampling bias as the presence data of the focal species (Phillips *et al*., 2009), but its performance depends on the range of environmental preferences of the species included in the target group (Botella *et al*., 2020). More recent approaches to modelling presence‐only data, by integrating them with any available presence–absence data (Fithian *et al*., 2015), may help minimise some of these biases.

### (2) Estimating trends in species abundances

Models to estimate species' trends tend to be descriptive: spatial variation is modelled by including site identity (as a fixed or random term) while any temporal trend is modelled as a simple year effect, either as a linear function, spline function or as a factor (Amano *et al*., 2012; Bled *et al*., 2013). Drivers of the trend are not explicitly modelled when the goal is simply to estimate the mean long‐term trend. As such, broader inferences about the estimated mean trend are based on the assumed representativeness of the sampled sites, or prior knowledge of sampling unit inclusion probabilities (see design weights discussed in Section IV.2). Relying on the representativeness of the sampling design is the most traditional approach to survey sampling (Smith, 1976) and the one typically taken by official governmental surveys using some form of random sampling (van den Brakel & Bethlehem, 2008). This approach has the advantage of avoiding complex assumptions in the statistical analysis (Buckland *et al*., 2012) and is perhaps also easier to analyse and communicate to stakeholders and laypersons.

**Fig. 3:**
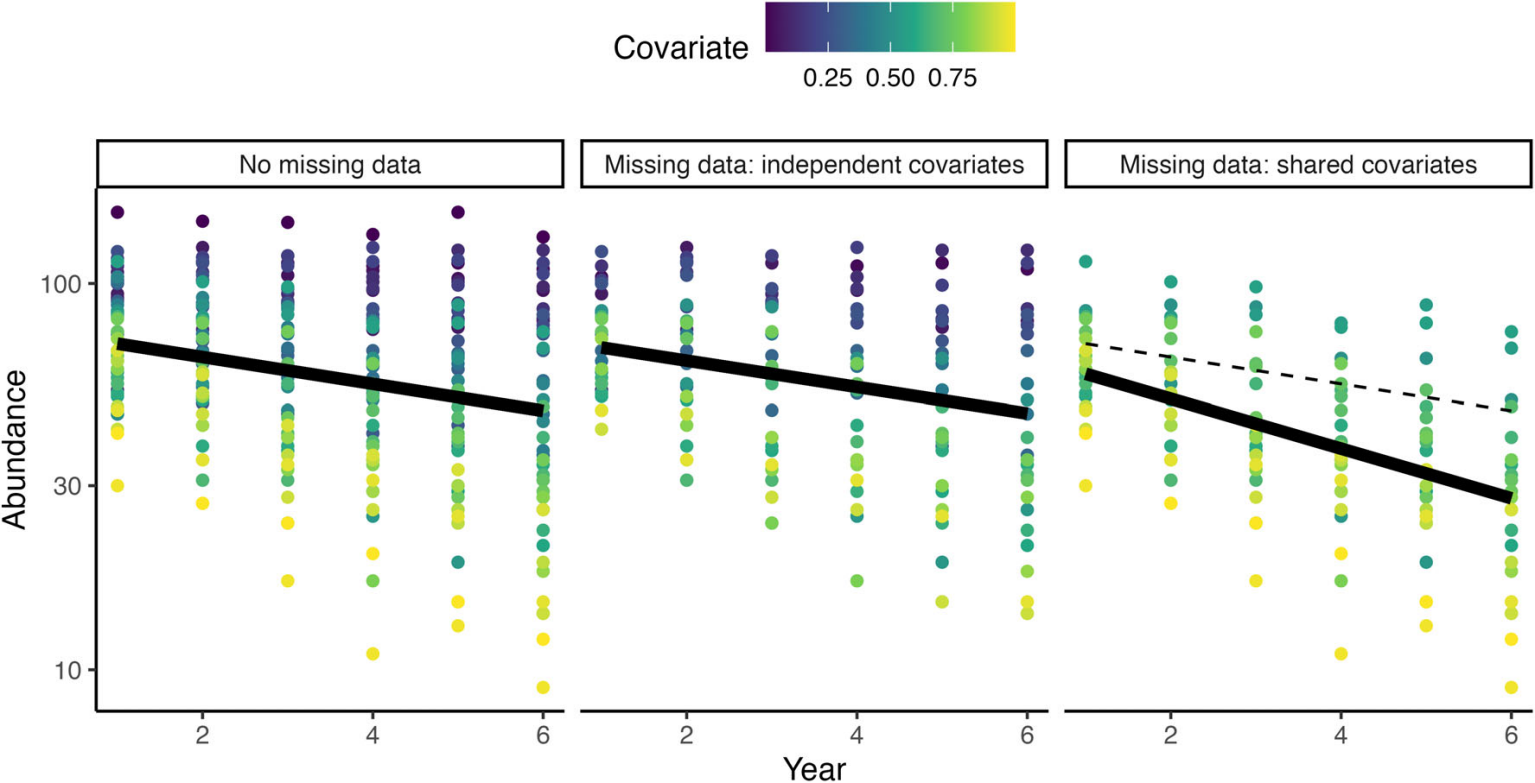
The impacts of different missing data mechanisms on trend modelling. We use a hypothetical scenario in which a mean trend model is fitted to data sets that vary in their missing data mechanism. We assumed a scenario of 50 sites that varied in an environmental covariate affecting species trends (trends were stable or even increasing at low values of the covariate and declining at increasingly high values of the covariate). When the probability of sampling a site was independent of the covariate driving species trends [i.e. a “missing completely at random” (MCAR) pattern – there are fewer points in each year in the middle panel, but they are a random set of those in the left panel, that is the covariate affecting sampling probability was a different and uncorrelated covariate to the one affecting species], the overall mean trend (estimated by the year effect in a generalised linear mixed‐effect model that also included a site random effect) was similar with (middle panel) and without (left panel) missing data. By contrast, when the same covariate affected both species' trends and sampling probability, leading to less sampling in sites with low values of the covariate [notice there are fewer blue points in the right panel – a “missing not at random” (MNAR) pattern], the overall mean trend was downward biased with missing data (right panel) compared to the scenario of no missing data (shown by the dashed black line and in the left panel).

Simple trend models may, however, lead to biased trend estimates when data gaps are not MCAR. We illustrate this in a simple simulation in which site‐level species trends were assumed to depend on a site‐level covariate, for example urban cover (Fig. 3). We assumed sites were sampled either with a probability affected by an independent covariate (Fig. 3 middle panel) or with a probability affected by the same site‐level covariate affecting species trends (Fig. 3 right panel), a scenario already identified in some monitoring schemes (Buckland & Johnston, 2017). We estimated the mean trend using a simple mixed‐effect model including site and year. The results show that when an independent covariate affected sampling, the trends were unbiased, but when the site‐level covariate affected both sampling and species' trends, the trends were biased (Fig. 3). In real‐world situations, many factors will simultaneously influence the trend of a species, but this simple simulation highlights the potential for bias caused by shared covariates. Since the specific causal covariates driving species trends or sampling probability are not included in the commonly used descriptive trend models, trend analyses are liable to be affected by MNAR. Without conditioning on the covariates involved, trend estimates might be underestimated if missing data are more common in regions where species trends are more strongly declining or overestimated if missing data are more common in regions where species are stable or increasing (Fig. 3) (Bowler *et al*., 2022; Buckland & Johnston, 2017).

## IV. MISSING DATA SOLUTIONS

**Fig. 4:**
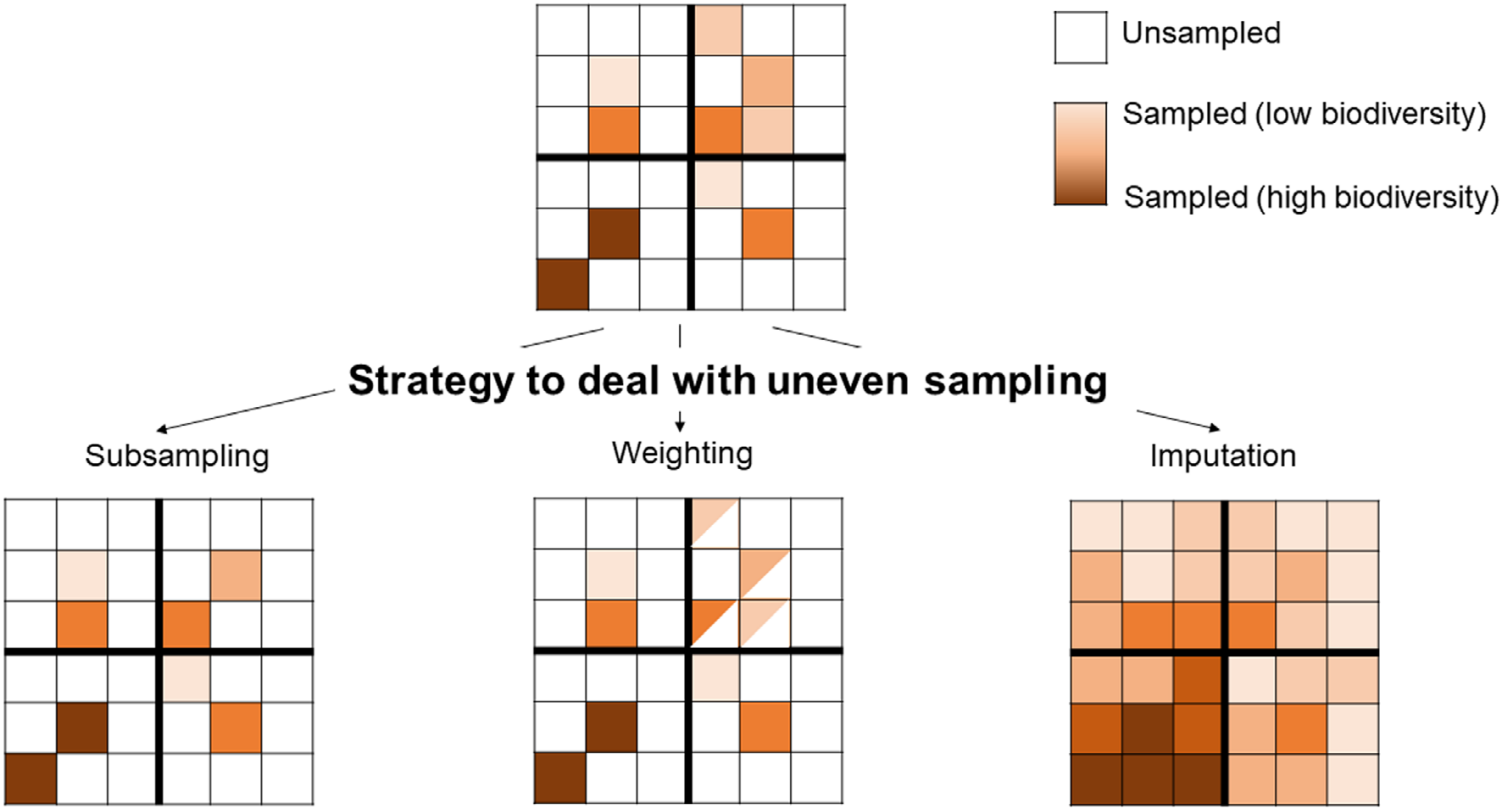
Visualisation of contrasting approaches to deal with data gaps. We focus on spatial gaps to illustrate the possible approaches, but the ideas apply to other types of data gaps (Fig. 1). In the top panel, the landscape is divided into four quarters (e.g. representing different habitats or geographic regions). One quarter (top right quarter) has been sampled more (four sampling sites) than the others (two sampling sites each). The bottom panel shows possible solutions. In random subsampling (bottom left), two sites are randomly subsampled from the oversampled quarter to create a data set with an even sampling coverage across quarters. In weighting (bottom middle), data from the oversampled quarter are down‐weighted in the statistical model so data from all quarters similarly influence the modelled results. In imputation (bottom right), missing values at unsampled sites are imputed based on the spatial pattern in the data and/or environmental covariates, and summary parameters are calculated based on both predictions at sampled and unsampled sites. In subsampling and weighting, the aim is to improve the representativeness of the sample for statistical inference at the population level. In imputation, the aim is directly to predict population‐level values.

A broad range of methods to deal with missing data have been used in ecology (Hossie, Gobin & Murray, 2021; Nakagawa & Freckleton, 2008; Lopucki *et al*., 2022). Many solutions are particularly relevant when data are missing in both response and predictor variables. Here, we focus on the typical scenario in biodiversity modelling of missing data only in the response variable (i.e. in the biodiversity data) since predictors typically used in large‐scale modelling tend to have no or few gaps (e.g. site identity or environmental data products derived from remote sensing). We organise solutions into three groups – subsampling, weighting and imputation (Fig. 4) – which have been tested to varying degrees already with both structured and unstructured biodiversity data (Table 2). Most solutions to deal with missing data are only appropriate for MCAR or MAR missingness, when data are available on the key covariates affecting sampling to be included in the analysis. MNAR is the most challenging class of missing data to deal with in statistical modelling, so we deal with MNAR separately in Section VI.

**Table 2:** Example applications of the solutions to deal with data gaps within biodiversity data.

Type of data gaps	Typical approaches
Within‐year	Sometimes imputed e.g. spline terms to smooth over seasonal variation in sampling times during the flight period of butterflies (Dennis *et al*., 2016; Schmucki *et al*., 2016)
Annual	Sometimes imputed e.g. generalised linear models to impute gaps based on mean site and year effects, optionally allowing for habitat differences, e.g. used in TRIM abundance indices (Lehikoinen *et al*., 2016)
Spatial	Often ignored, but occasionally weighting by geographic regions (Bled *et al*., 2013) or imputed (Breivik *et al*., 2021) or reduced by subsampling (Johnston *et al*., 2021).

### (1) Subsampling

The “Big Data Paradox” highlights that there can be trade‐offs between data set size and data set quality (Bradley *et al*., 2021; Meng, 2018). Small data sets can be preferable to large data sets if they are more representative and less heterogeneous (Bayraktarov *et al*., 2019). Based on such thinking, some studies have proposed “reverse engineering” structure in biodiversity data by filtering data points (Rapacciuolo, Young & Johnson, 2021). Part of this reverse engineering has attempted to deal with spatial biases; for instance, by spatially subsampling data to reduce the unevenness of sampling effort across the landscape (Steen *et al*., 2021; Matutini *et al*., 2021; Steen, Elphick & Tingley, 2019; Boria *et al*., 2014; Robinson *et al*., 2020). This has been tested on, for instance, the semi‐structured data compiled by eBird (Johnston *et al*., 2021). Typically, subsampling is done using geographic covariates or spatial units, such as grid cells, rather than using environmental covariates that are assumed to have a causal link with either sampling or species. Some have also applied this approach to reduce the effects of temporal changes in sampling effort (Hof & Bright, 2016; Zbinden *et al*., 2014), although not always successfully (Callcutt, Croft & Smith, 2018). Subsampling could also be used to balance the amount of data across a single or multi‐dimensional environmental gradient; essentially stratified sampling of the original sample (Meng, 2022; Nunez‐Penichet *et al*., 2022). Recent class balancing approaches have been developed to ensure that important detections for rare species are not lost during the subsampling process (Robinson *et al*., 2020; Steen *et al*., 2021; Gaul *et al*., 2022).

### (2) Weighting

Weighting is a common practice in survey analysis, especially in the social sciences (Li *et al*., 2013; Seaman & White, 2013; Raghunathan, 2004; Valliant, Dever & Kreuter, 2018). Weighting can serve different purposes, including reducing the impact of confounding variables when the goal is to estimate the causal effect of an intervention. But weighting can also be used to deal with missing data that are not MCAR. For instance, weighting has been used to reduce selection bias caused by participant non‐response in surveys (Seaman & White, 2013), but it is less often used to account for data gaps in biodiversity modelling (Boyd, Powney & Pescott, 2023; Aubry & Francesiaz, 2022).

Different types of weights have been used in the analysis of biodiversity data, especially to deal with spatial gaps: (1) design weights; (2) non‐response weights (or sampling weights) and (3) population weights. Each form of weighting is intended to improve sample representativeness of some target population but vary in terms of whether or not the weights derive from the sampling design and the dimension of representativeness under consideration. Design weights are based on the study sampling design and assumed to be known with certainty, and hence are only relevant for structured monitoring schemes with a sampling design. For instance, in many national bird breeding schemes, the design weights are based on the geographic strata that underlie a random stratified study design (Buckland *et al*., 2012). Non‐response weights can be used to account for unplanned missing data in structured schemes (Frair *et al*., 2004) or variation in sampling effort in unstructured schemes (Johnston *et al*., 2020; Hefley *et al*., 2013). In both cases, the non‐response weights must be estimated based on the available data and hence differ from design weights since they cannot be known with certainty. Population weights are primarily used in the calculation of supranational/international indicators in which estimates from national surveys are combined (e.g. farmland or woodland bird indicators; Gregory *et al*., 2005). For these indicators, populations weights are used to give greater weight to data from regions/countries that harbour a larger proportion of the species' total population, when calculating the overall mean.

Non‐response weights are usually the most difficult to include since they are not known *a priori* and need to be estimated. Predictive models (e.g. random forest models) have been used to predict the probability that a site is sampled based on a set of covariates (e.g. land cover or climate, or accessibility) available across all sampled and unsampled sites, with the inverse of these probabilities used as weights (Little *et al*., 2022; Johnston *et al*., 2020). Alternatively, post‐stratification (for categorical covariates or subgroups termed strata), or more generalised calibration approaches (allowing both continuous and categorical covariates), can be used, which adjust the weight given to each data point until the joint or marginal distributions of covariate values in the observed sample matches those for the population. For instance, when estimating the occupancy change of a plant species in the UK, Boyd, Stewart & Pescott (2024) used data on elevation – a factor affecting both sampling and species occupancy – to upweight data from under‐sampled high‐elevation regions and produce more accurate estimates of the species distribution size at different time points. In both cases, weighting can cause problems when there are regions within the target population with close to zero probability of being sampled, which could lead to some data points having extremely large weights. In this case, weights may need to be redefined, for example by coarsening the covariates used to define the weights so that all strata have some probably of being sampled, or by truncating weight values so that extreme weights are not produced (Battaglia, Hoaglin & Frankel, 2009). Another approach that can help deal with small sample size in some strata is so‐called “Mr P” analysis (= Multilevel Regression with Post‐stratification). With this approach, variables for the sampling strata are included as random effects in a multilevel regression/mixed‐effect model, so that there is partial pooling of information across strata, before the model predictions for each strata are reweighted for representativeness of the target population (Gelman, 2007; Authier, Rouby & Macleod, 2021).

The most appropriate approach to using weighting is likely to be question and taxon specific, varying with how much the species range extends across the region of interest. For example, when estimating trends in the total population size of a species, it might not be important to upweight under‐sampled regions if those regions overlap with where a species is rare, or even absent. If, however, the goal is to estimate trends in average site‐level population trends of a species, then it would be important to up‐weight data from under‐sampled regions, even from where the species is rare. For instance, in the UK bat monitoring scheme, data are weighted to allow for the different sampling rates across England, Scotland and Wales in proportion to the ratio of non‐upland area to number of sites surveyed for the relevant country (Bat Conservation Trust, 2023). However, this weighting is not applied to range‐restricted species, such as the serotine bat, *Eptesicus serotinus* that is only found in southern England.

### (3) Imputation

Imputation involves replacing missing values in a data set with plausible estimates. A range of imputation procedures have been developed, which can fill gaps in both response and predictor variables (Carpenter & Kenward, 2012). Imputation is probably the most flexible and widely used approach to account for missing data across ecology and beyond. In biodiversity modelling, missing values are more often concentrated in the response variable (i.e. the biodiversity value), so imputation here can be equated with making model predictions at unsampled sites and times.

Imputation is already in use in species trend monitoring, especially to account for within‐year and annual data gaps (Table 3). Early approaches used chain indices or route regression (Ter Braak *et al*., 1992) or the Underhill index, using an expectation‐maximisation algorithm, designed for waterbirds (Underhill & Prysjones, 1994; Rehfisch *et al*., 2003). A range of further model‐based approaches have been developed that fill data gaps using mean effects of site and year, for example to fill annual gaps using TRIM/birdSTATs, commonly used for bird indices (Lehikoinen *et al*., 2016); or using temporal splines, for example to fill seasonal gaps in butterfly sampling (Schmucki *et al*., 2016; Dennis *et al*., 2016) or using ecological covariates (Dakki *et al*., 2021). A Bayesian framework can be especially useful for dealing with missing values in the response since they are naturally imputed with a full probability distribution during model fitting, for example with Just Another Gibbs Sampler (JAGS) or NIMBLE. For instance, Bayesian occupancy‐detection models have been used to analyse opportunistic species observations from citizen science, with annual data gaps at each site imputed before the predicted annual proportion of occupied sites is calculated (Outhwaite *et al*., 2019). The flexibility of Bayesian models means they could also incorporate expert knowledge as priors to help fill data gaps (Johnson *et al*., 2023).

**Table 3:** Summary of the pros and cons of each approach to deal with missing data in biodiversity monitoring.

Solution	Pros	Cons
Subsampling	– arguably the simplest approach, especially for spatial gaps – already a routine feature of many species distribution modelling protocols – aligns with rarefaction approaches used in community ecology	– could mean excluding a large amount of data, which may be unacceptable for citizen science and engaging/retaining volunteers – most protocols focus on a single dimension (e.g. filtering by geographic region) – more complex to implement when gaps are multi‐dimensional or temporally varying
Weighting	– standard practice to deal with sample unrepresentativeness in other disciplines, especially social sciences	– less commonly applied in ecology – diverse range of possible weighting techniques (Valliant, 2020; Boyd *et al*., 2024) but little guidance available for ecologists to decide which approach to use
Imputation	– suitable approach if missing data are within the environmental covariates as well as within the biodiversity response – offers the promise to generate the continuous space–time data cubes of the Essential Biodiversity Variable framework (Kissling *et al*., 2018; Jetz *et al*., 2019).	– requires a good understanding of the ecological system to predict the missing biodiversity values – inefficient when the number of unsampled sites/times is large if the goal is only to estimate mean abundance or occupancy

While imputation is already used to deal with annual and within‐year gaps, it has been used less often to deal with spatial gaps when the focus is modelling change over time in species' abundances or occurrences. An exception is studies of changes in species' range sizes using distribution models that predict the full distribution of a species at multiple time points before change is assessed (e.g. Grattarola, Bowler & Keil, 2023). Monitoring schemes with large spatial coverage (e.g., eBird) are also beginning to use models to predict spatio‐temporal patterns of abundance change across whole countries (Fink *et al*., 2020). In these cases, statistical models of the effects of environmental covariates and/or spatial structure are used to make predictions at unsampled sites (Bush *et al*., 2017; Ver Hoef *et al*., 2021; Breivik *et al*., 2021). Geostatistical methods, such as kriging, also offer a range of interpolation methods for spatial data, which are especially useful when there is a strong spatial autocorrelation (Ballesteros‐Mejia *et al*., 2013; Kreft & Jetz, 2007; Lin *et al*., 2008).

## V. PRO AND CONS OF EACH SOLUTION

All of the above‐mentioned approaches have the potential to reduce the bias in parameter estimates associated with data gaps but differ in complexity, scope and typical practice (Table 3) (Little *et al*., 2022; Collins *et al*., 2001). Moreover, while we separated the methods into three categories for convenience, their distinctions are not absolute. For instance, subsampling essentially assigns included data points a weight of 1 and the remainder a weight of 0. Often, but not always, the reduction in bias due to application of the above solutions comes at a cost of increased parameter uncertainty: the classic bias–variance trade‐off (Hefley *et al*., 2013). This is because subsampling directly reduces the sample size; weighting can reduce the effective sample size; and imputation adds uncertainties *via* predictions at unsampled points. But this trade‐off does not always apply; for instance, post‐stratification can lead to the dual benefits of reduced bias and increased precision depending on the choice of covariates (Little & Vartivarian, 2005).

Covariates used to account for data gaps are often called “auxiliary variables” (Little *et al*., 2022), which are typically not of central interest to the scientific questions but are used to adjust for missing data. The general recommendation from the missing data theory and survey sampling literature is to be generous when deciding on auxiliary variables, considering those relating to the missingness (i.e. sampling effort in the context of biodiversity data gaps) to reduce bias, and to the biodiversity outcome to reduce variance (Collins *et al*., 2001; Caughey *et al*., 2020). It is worth noting, however, that selecting auxiliary variables purely on the hypothesised strength of the correlation can increase bias in some circumstances (Thoemmes & Rose, 2014), and a safer strategy is to select covariates *a priori* based on causal reasoning (Mohan & Pearl, 2021). When auxiliary variables are related to both the biodiversity outcome and the pattern of missingness, weighting approaches can reduce bias and improve precision (Little & Vartivarian, 2005). The success of any of the solutions, hence, critically depends on the choice of auxiliary variables (Little *et al*., 2022). A recent study testing the use of weighting approaches to account for spatial biases in a reasonably well‐understood ecological system found that the selected auxiliary variables had only limited success in mitigating bias (Boyd *et al*., 2024), suggesting that the limiting factor in accouting for bias often may be defining the right auxillary variables.

**Fig. 5:**
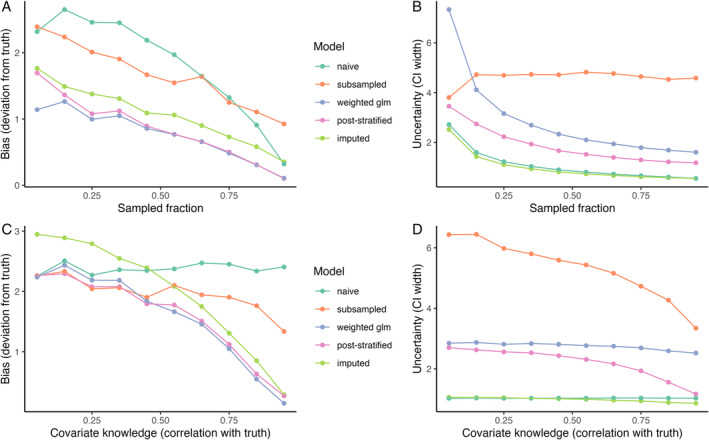
The ability of missing data solutions to adjust for bias in biodiversity data. We assumed a landscape of 400 cells and that a cell‐varying covariate affected both species abundance and the likelihood of a cell being sampled. In A and B, we varied the fraction of the cells that were sampled. In C and D, we varied the correlation between the true covariate and the covariate available for analysis, as measure of our knowledge (correlation of 1 = perfect covariate and knowledge). The models used to estimate the parameter of interest (mean abundance) were: naive [no correction, Poisson generalised linear model (GLM)]; subsampled (cells were subsampled along the covariate gradient), weighted (two methods: weighted GLM and post‐stratification, using postStratify in the *survey* package) and imputed (using Just Another Gibbs Sampler, or JAGS, in which missing values were set as “NA” in the response). Points in A and C show the mean bias (difference between model prediction and truth – note the true mean value was 7.3) while B and D show the mean width of the confidence intervals (CI) of the mean abundance estimate across 100 independent runs. In A and B, covariate knowledge was fixed at a correlation of 0.75; while in C and D, the sampling proportion was fixed at 0.35.

We illustrate some of these challenges and the application of each potential solution with a toy example of an abundance data set with missing values (Fig. 5). We simulated a landscape in which a covariate (for example representing habitat quality) affected both species abundance and the likelihood of a site being sampled. The analysis aimed to estimate the mean abundance of the species across all sites in the landscape. We varied the total fraction of sites that were sampled and the degree of knowledge available on the covariate affecting sampling/the species (modelled as the correlation between the covariate involved in the data‐generation process and the covariate available to the modeller). We compared subsampling, weighting and imputation, which all used the available covariate data for adjustment. For subsampling, we subsampled one site at random for each value of the covariate. For weighting, we compared two approaches: first, fitting a generalised linear regression model with cell‐specific weights (inverse of sampling probability) using model‐robust variance estimators that take into account the weighting of the observations, and second, using post‐stratification to weight data so the covariate distribution of the sampled data matched that of the population – in this case the unique covariate values were treated as separate sampling strata (Valliant *et al*., 2018). For imputation, we fitted a Bayesian generalised linear regression model (using JAGS) in which missing values were set as “NA” in the response and were imputed based on the estimated effect of the covariate.

The results show that all methods do better, in terms of reducing bias, than a naive approach that did not attempt to account for missingness in the estimation of the mean abundance (Fig. 5A, C). Subsampling performed the worst, and weighting the best. Post‐stratification tended to perform slightly less well (i.e. led to higher bias) when the sampling fraction was low, when the number of missing values was high (Fig. 5A). This latter pattern was because the sample did not contain all the habitat quality values found in the population, meaning there were no available data to upweight in under‐sampled regions. All models performed less well, shown by higher bias, as the available covariate became a weaker proxy of the true driving covariate (lower correlation with the truth; Fig. 5C), especially imputation. In terms of uncertainty of the parameter estimates (measured as the width of the confidence interval), attempts to adjust for bias usually increased the width of the confidence intervals (Fig. 5B, D) – subsampling led to the greatest increase in uncertainty (explained by reduced sample size), while weighting added intermediate levels of uncertainty. For post‐stratification, the increase in uncertainty was minimal when the covariate was a good proxy (Fig. 5D), which is a pattern noted elsewhere (Little & Vartivarian, 2005). In this simple example, imputation led to a similar uncertainty – in terms of width of the confidence interval – as the naïve approach, partly because we assumed a relatively small and well‐sampled system. In further simulations, we found that imputation performed less well when there were additional covariates affecting species abundance and these covariates were not modelled, highlighting the importance of understanding the ecological system for imputation (see online Supporting Information, Fig. S1). We do not intend this simulation to be exhaustive – rather to highlight the potential ways in which the availability of data and degree of knowledge about the factors causing bias affect any attempts to account for missing data. We point the reader towards some useful R packages and functions in Table S1.

## VI. DEALING WITH MISSING NOT AT RANDOM

Dealing with MNAR is more challenging than dealing with the other classes of missing data (Little & Rubin, 2019). In this case, missingness is directly associated with unavailable data, which could be either because sampling is affected by the missing biodiversity values or important covariates that are not known to be important and/or are not measured or measurable. This makes MNAR especially difficult to diagnose [but see Conn *et al*. (2017) for suggestions] and model, since possible auxiliary variables to adjust for the data gaps are not available. MNAR can arise through several mechanisms in biodiversity monitoring data.

MNAR can be an outcome of preferential sampling – more intense sampling effort where the species is expected (Diggle, Menezes & Su, 2010; McClure & Rolek, 2023) – leading to more missing values in places where the species is rare or absent. Preferential sampling can arise, for instance, if observers visit a location specifically to observe a species that others have observed there before (Laney *et al*., 2021; Pennino *et al*., 2019). Preferential sampling can also be a planned sampling strategy (Alessi *et al*., 2023). For rare species, preferential sampling can be chosen when the goal is to estimate species detection probability and account for imperfect detection, since sufficient observations of the species can only be achieved by sampling where they are found (Specht *et al*., 2017). Similarly, it can be optimal to expend greater sampling effort where the species is common if the goal is to estimate trends in the total population size, since regions where the species is scarce are less important for the overall trend. For organisms associated with specific habitats, such as wetland species or colonial seabirds, dedicated structured monitoring schemes target their habitats (McClure & Rolek, 2023). In such schemes, missing data outside of these core habitats are not considered part of the target population.

Typical approaches to modelling data allowing for MNAR are selection models (Heckman, 1979) and pattern‐mixture models (Little, 1993). Both model the joint distribution of the data and the data availability, but differ in how these processes are decomposed. Both also require making strong assumptions about the missing data mechanism but can be used to explore the consequences of plausible missing data mechanisms as sensitivity analyses (Little, 1995). In the ecological literature, approaches to deal with preferential sampling have also involved jointly modelling the sampling intensity, the biodiversity value at sampled points and the dependence between them, such as using marked point process models (Conn *et al*., 2017; Pennino *et al*., 2019; Laxton *et al*., 2023). Meta‐analyses often face similar MNAR problems, caused by publication bias when data are missing according to values of the data itself. In meta‐analysis, similar sensitivity analyses, including selection models and the trim‐and‐fill method, have been proposed to test the robustness of model predictions to possible assumptions about missing data (Maier, VanderWeele & Mathur, 2022; Sutton *et al*., 2000). Another approach to inference in a MNAR scenario is to use instrumental variables, i.e., variables that affect the probability of sampling/data availability but are independent of the biodiversity variable of interest (Tchetgen & Wirth, 2017; Bailey, 2023); however, the challenge is to identify such variables.

## VII. GENERAL GUIDELINES FOR DEALING WITH BIODIVERSITY DATA GAPS

Our review highlights the potential value of “missing data thinking” when analysing biodiversity data. We argue that MCAR data gaps are unlikely in most biodiversity data contexts because at least some of the known factors affecting sampling probability, especially accessibility, urban land cover and human population density, overlap with those affecting species. This means that researchers will need to consider whether and how they deal with data gaps in their analysis. While it is premature to make very specific guidelines, we summarise here some of the considerations needed when dealing with data gaps in biodiversity data at different stages of data collection, analysis and reporting.

### (1) Study design

For new monitoring schemes, planned data gaps that deviate from MCAR (i.e. a random sample) can be seen as opportunities rather than challenges since solutions are available to deal with missing data, provided that sampling inclusion probabilities are known. Indeed, planned data gaps are already used in schemes with a spatially stratified sampling design, often in relation to sampling probabilities of different geographic regions. In other fields, beyond monitoring, intentionally missing data has been proposed for ethical or practical reasons (e.g. Noble & Nakagawa, 2021; Herrera, 2019). In citizen science, planned data gaps could help increase uptake and avoid participant fatigue, especially caused by collecting difficult data. For instance, the UK Breeding Bird Survey includes an “upland rovers” component in which the standard protocol is modified to allow for fewer visits to remote sites, with the long‐term aim of increasing spatial coverage of the data (Darvill *et al*., 2020). Alternative study designs, such as wave missingness or a rotating panel design (Nielsen *et al*., 2009; Little & Rhemtulla, 2013) explicitly incorporate planned data gaps (e.g. years when a site is not planned to be surveyed) and may similarly increase the sustainability of long‐term monitoring for some taxa or regions with few willing participants. But such an approach has to balance the cost of increased study design complexity and potential implications for the range of questions that can be addressed.

For existing monitoring schemes, data gaps may be filled, where possible, by promoting data collection in regions that represent sampling priorities – either because they lack data or because they are dissimilar to sampled regions. Within citizen science projects, there is evidence that participants can be nudged to collect more data in regions identified as sampling priorities (Callaghan *et al*., 2019, 2023). Previous studies have identified sampling priorities in different ways; for instance, based on the expected influence of a data point (Callaghan *et al*., 2019) or predictions based on species distribution models (Chiffard *et al*., 2020). A similar targeting of effort may be used in synthesis studies that compile data from independent studies. In this case, efforts to mobilise data may be targeted towards under‐represented sites/times, or those with the most uncertain predictions according to models of the existing data.

### (2) Evaluating and reporting missingness

Developing a causal model of the factors affecting sampling probability and species [e.g. using a directed acyclic graph (DAG) to visualise the hypothesised causal links] can be a useful first step to identify the covariates linked to both sampling probability and species occurrence or abundance (Mohan & Pearl, 2021; Hughes *et al*., 2019). As far as possible, data should then be collected on these covariates. Statistical models can be used to test whether covariates that are associated with missingness are also associated with the species, although of course the latter is only possible in the sampled data. Unplanned missingness in structured monitoring schemes could be investigated by disseminating follow‐up surveys to participants to determine their reasons for missed surveys. Follow‐on data collection, for example with paid surveys, or targeted citizen science, in regions or times of missing data could also help understand whether there are fundamental differences in species occurrence/abundance between the original data set and the extended data set.

Missingness, and how it is dealt with, is often not clearly reported in species trend analyses. Some reporting frameworks for missing data have been developed for other disciplines (Lee *et al*., 2021). Such frameworks are in their early stages in ecology, but an approach has been proposed recently (Boyd *et al*., 2022) that builds on the “risk of bias” tools used in other fields, especially in systematic reviews in medicine (Babic *et al*., 2019). At a minimum, we propose that missingness can be reported in terms of the proportion of sampling units that are spatial, annual and within‐year gaps, and the number of unplanned gaps for structured monitoring schemes (Fig. 1). But also important are summaries or visualisations of the distributions of environmental covariate values in sampled and non‐sampled times/sites to highlight potentially important differences between the sample and target population of inference.

### (3) Modelling to account for data gaps

The impact of data gaps will depend on multiple factors: their frequency and contiguity; how well data gaps are understood; whether the factors affecting missingness are independent of the factors affecting species and species abundance itself; the ecological questions being asked and which covariates are available and included in the analysis. Because of this, the potential impacts of missingness and possible solutions should be considered for each species–question–data set combination. A data set *per se* is not biased. Subsampling, weighting and imputation all have potential to reduce bias caused by data gaps. Weighting is probably the most under‐used in ecology and could be applied more often, especially to account for spatial gaps when the goal is estimating overall means or mean trends in abundance or occupancies. Imputation methods offer the potential to fill in spatio‐temporal gaps to generate the space–time data cubes underlying the Essential Biodiversity Framework (Kissling *et al*., 2018), but their success depends on the ability to model variation in the biodiversity response. If bias is expected to be strong, but the causes are not fully known or relevant covariate data not fully available to adjust for it, the broader implications that can be drawn from a model of the data become difficult to communicate. Sensitivity analysis could help explore how different assumptions of the missingness would affect model interpretation and the robustness of conclusions (Little, 1995; Leurent *et al*., 2018). Alternatively, it might be sensible to redefine the target region of interest to a region with fewer data gaps so that the sampled data are more representative of the target population. If this is not possible due to wide data gaps, a final option might be to revise the generality of the study question to make explicit the limits of information within the sampled data.

## VIII. CONCLUSIONS

(1) Biodiversity data sets containing information on species' occurrences and abundances are rapidly growing in size, but data gaps are not necessarily closing. Nonetheless, big biodiversity data sets are invaluable for a broad range of basic and applied questions, and increasingly for policy‐relevant questions about the status and trends of biodiversity at large scales. Heterogeneity in sampling efforts – whether by volunteer citizen scientists or contracted surveyors – creates different types of data gaps in the available data. Such data gaps are among the biggest hindrances to making use of these growing data sources for large‐scale inferences about biodiversity patterns.
(2) We show how “missing data thinking” can help decide whether a data gap is problematic in a given context and provides directions on possible solutions. We show that an important determinant of bias is whether factors affecting sampling effort are correlated with those affecting species: shared covariates affecting sampling effort and species occurrence or abundance have the potential to lead to biased analyses if not taken into account.
(3) Multiple approaches are available to account for missing data but they depend on knowledge and availability of covariates associated with missingness. A lack of training for ecologists in commonly employed approaches in other disciplines has meant there are few standard practices in ecology to deal with gaps. We highlight multiple methods that are ripe for comparison across different ecological problems.
(4) At the same time, statistical solutions can only go so far, closing data gaps with more coordinated data collection across stakeholders in biodiversity and environmental monitoring is also important to advance predictions of the state of, and trends in, biodiversity.

## Supporting information


**Fig. S1.** The ability of missing data solutions to adjust for bias in biodiversity data: extended analysis with additional covariates affecting the biodiversity response.
**Table S1.** Selected R tools that can help with missing data problems and their potential application for use in biodiversity research.

## Data Availability

R script for the example solution simulations (Figs 5 and S1) can be found at: https://github.com/bowlerbear/dataGaps.
