# Supplementary material for: Treating gaps and biases in biodiversity data as a missing data problem
Source: Biol Rev Camb Philos Soc. 2024 Aug 8;100(1):50–67. doi: 10.1111/brv.13127 (PMC11718628; doi:10.1111/brv.13127)
Supplement: Supplementary file 1 — Fig. S1. The ability of missing data solutions to adjust for bias in biodiversity data: extended analysis with additional covariates affecting the biodiversity response. Table S1. Selected R tools that can help with missing data problems and their potential application for use in biodiversity research. [file BRV-100-50-s001.docx]

Supporting Information for:

**Treating gaps and biases in biodiversity data as a missing data problem**

Diana E. Bowler, Robin J. Boyd, Corey T. Callaghan, Robert A. Robinson, Nick J. B. Isaac and Michael J. O. Pocock

Correspondence to: diana.e.bowler@gmail.com


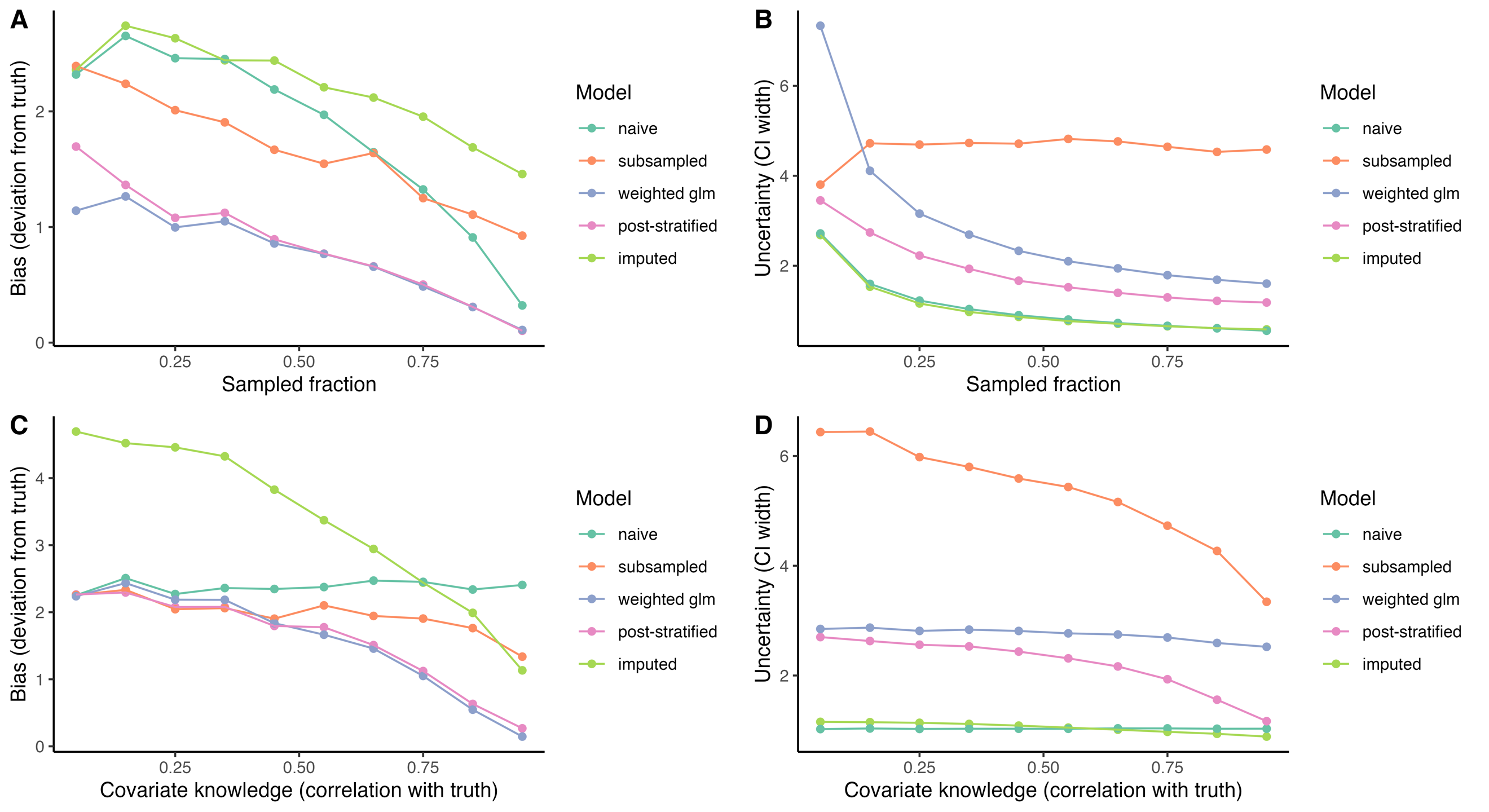


**Fig. S1.** The ability of missing data solutions to adjust for bias in biodiversity data: extended analysis with additional covariates affecting the biodiversity response. We assumed a landscape of 400 cells and that a cell-varying covariate affected both species abundance and the likelihood of a cell being sampled. In A and B, we varied the fraction of the cells that were sampled. In C and D, we varied the correlation between the true covariate and the covariate available for analysis, as measure of our knowledge (correlation of 1 = perfect covariate and knowledge). The models used to estimate the parameter of interest (mean abundance) were: naive [no correction, Poisson generalised linear model (GLM)]; subsampled (cells were subsampled along the covariate gradient), weighted (two methods: weighted GLM and post-stratification, using postStratify in the *survey* package) and imputed (using Just Another Gibbs Sampler, or JAGS, in which missing values were set as ‘NA’ in the response). Points in A and C show the mean bias (difference between model prediction and truth – note the true mean value was 7.3) while B and D show the mean width of the confidence intervals of the mean abundance estimate across 100 independent runs. In A and B, covariate knowledge was fixed at a correlation of 0.75; while in C and D, the sampling proportion was fixed at 0.35. In contrast to Fig. 5 in the main text, here we assumed that species abundance was affected by an additional variable that was not included in any of the subsequent analyses.

**Table S1.** Selected R tools that can help with missing data problems and their potential application for use in biodiversity research.

| **R packages** | **Applications** | **Useful functions** |  |
| --- | --- | --- | --- |
| *Exploring missing data* | | | |
| [naniar](https://cran.r-project.org/web/packages/naniar/vignettes/getting-started-w-naniar.html) | visualizing/exploring the missing data pattern | *mcar_test* – Little's missing completely at random (MCAR) test  *vis_miss* – plot the missing data for all variables |  |
| [occAssess](https://onlinelibrary.wiley.com/doi/full/10.1002/ece3.8299) | measure of the potential for bias in taxonomic, temporal, spatial, and environmental dimensions | *assessEnvBias* – assess whether data are sampled from a representative portion of environmental space in the spatial domain of interest  *assessSpatialBias* – assess whether data resemble a random distribution in the geographic space of interest for inference  *assessSpatialCov* – assess whether a representative portion of the spatial domain of interest has been sampled and whether the same portion of geographic space has been sampled over time |  |
| [sampbias](https://onlinelibrary.wiley.com/doi/full/10.1111/ecog.05102) | a Bayesian approach to estimate how sampling rates vary as a function of proximity to one or multiple bias factors | *calculate_bias* – calculate the bias effect of sampling bias due to geographic structures, such as vicinity to cities, airports, rivers and roads |  |
| *Subsampling* | | | |
| base | Base R functions | *sample* – sample data with predefined inclusion probabilities specified with the prob argument |  |
| [sampling](https://cran.r-project.org/web/packages/sampling/index.html) | draw random samples using different sampling schemes | *balancedcluster* – selects a balanced cluster sample according to defined auxiliary variables  *strata* – stratified sampling with unequal probabilities |  |
| [spatialEco](https://cran.r-project.org/web/packages/spatialEco/index.html) | spatial data manipulation and modelling | *stratified.random* – creates a stratified random sample of an sp class object  *stratified.distance* – draws a minimum, and optional maximum constrained, distance sub-sampling |  |
| [spThin](https://onlinelibrary.wiley.com/doi/10.1111/ecog.01132) | spatial thinning of species occurrence records | *thin ­*– returns a data set with the maximum number of records for a given thinning distance |  |
| [terra](https://cran.r-project.org/web/packages/terra/index.html) | spatial data manipulation and processing | *spatSample* – sample a SpatRaster, SpatVector or SpatExtent object |  |
| *Imputation* | | | |
| [agTrend](https://besjournals.onlinelibrary.wiley.com/doi/full/10.1111/2041-210X.12231) | modelling regional trends with missing data | *mcmc.aggregate* – a zero-inflated, nonparameteric model with a definable observation model, augmenting missing values before calculating regional abundances |  |
| [INLA](https://www.r-inla.org/)/  [inlabru](https://sites.google.com/inlabru.org/inlabru) | fitting Bayesian models, especially useful for spatial models *via* its spatial mesh | *inla/bru* – fit a Bayesian model using Integrated Nested Laplace approximation  *predict* – draw predictions from the fitted model, where the prediction data frame can be a SpatialPointsDataFrame object |  |
| [LORI](https://cran.r-project.org/web/packages/lori/vignettes/getting_started.html) | imputation of missing count data | *lori* – impute missing count data using a large covariate set, including interactions, with a LASSO penalty |  |
| [mice](https://cran.r-project.org/web/packages/mice/mice.pdf) | multiple imputation by chained equations | *mice –* multiple imputation method that will generate plausible values for any missing data in the response and in any covariates |  |
| [Rjags](https://cran.r-project.org/web/packages/rjags/rjags.pdf)  [JAGS](https://mcmc-jags.sourceforge.io/)  [nimble](https://r-nimble.org/) | fitting Bayesian models allowing for missing values in the response | *Jags/runMCMC* – fitting Bayesian models allowing for imputation of missing values in the response during model fitting (options available for missing values in covariates too) |  |
| [rtrim](https://cran.r-project.org/web/packages/rtrim/index.html) | functions to calculate annual indices and trends of abundances | fit a GLM imputing missing values based on mean site and year effects, with optional covariates |  |
| *Weighting* | | | |
| [survey](https://cran.r-project.org/web/packages/survey/index.html)  [srvyr](https://cran.r-project.org/web/packages/srvyr/index.html) | range of functions for analysis of data from complex surveys, including fitting models with weights | *Svyglm* – generalized linear models with survey weights  *postStratify* – function for post-stratification to match the joint distribution of the variables of the population |  |
| [svrep](https://cran.r-project.org/web/packages/svrep/index.html) | analysis of replicate/boostrapped survey weights | *svyby_repwts* – compare estimates from different sets of weights |  |
| [twang](https://cran.r-project.org/web/packages/twang/index.html) | functions to estimate propensity scores and weights | *ps* – gradient boosted trees to predict non-response from covariates  *bal.table* – compare covariate values between sample and population |  |
